# Characterization of the intestinal microbial profile in mild cognitive impairment and Alzheimer's disease

**DOI:** 10.1590/1980-5764-DN-2025-0440

**Published:** 2026-08-21

**Authors:** Martin Medrano, Mar Pacheco-Herrero, Zunilda Borges, Johan de la Rosa Laureano, Jesabelle Dominguez-Garcia, Ruth Gil-Ventura, Gelanys Castro-Tejada

**Affiliations:** 1 Pontificia Universidad Católica Madre y Maestra, Vicerrectoría de Investigación e Innovación y Escuela de Medicina, Santiago de los Caballeros, República Dominicana.; 2 Pontificia Universidad Católica Madre y Maestra, Facultad de Ciencias de la Salud, Laboratorio de Investigación en Neurociencias, Santiago de los Caballeros, República Dominicana.; 3 Clínica Corominas, Servicio de Gastroenterología, Santiago de los Caballeros, República Dominicana.; 4 Westchester Medical Center, Department of Neurology, Valhalla, NY, USA.

**Keywords:** Alzheimer Disease, Cognitive Dysfunction, Gastrointestinal Microbiome, Dysbiosis, Dominican Republic, Enfermedad de Alzheimer, Disfunción Cognitiva, Microbioma Gastrointestinal, Disbiosis, República Dominicana

## Abstract

Alzheimer's disease (AD) and mild cognitive impairment (MCI) are major contributors to dementia, with growing prevalence in Latin America. Evidence suggests that gut microbiota alterations may influence neurodegeneration, but data on Hispanic population are lacking.

**Objective:**

Characterize gut microbiota in individuals with AD, MCI, and controls in the Dominican Republic, exploring clinical, demographic, and dietary associations.

**Methods:**

Prospective-translational study including 88 participants aged ≥60 years. Performed clinical, cognitive, and functional assessments. Stool samples analyzed using 16S rRNA gene sequencing. Bioinformatic processing using Quantitative Insights into Microbial Ecology Version 2 (QIIME2) and R. Alpha and beta diversity, taxonomy, and differential abundance were evaluated. Dietary influences were assessed using PERMANOVA.

**Results:**

No significant differences in alpha diversity (Shannon index 4–5, Simpson index 0.94–0.99, p>0.05) or beta diversity (p>0.05) were observed between groups. Firmicutes (51.9%) and Bacteroidota (34.1%) dominated the microbiota. Higher Desulfobacterota abundance in MCI and AD (0.54 and 0.61%, respectively, vs. 0.34% in controls; p<0.05). The Firmicutes/Bacteroidota ratio was lower in men with MCI (1.09) compared to controls and AD (1.70). MCI and AD were associated with increased levels of the genera Bilophila, Odoribacter, and Parabacteroides (p<0.05) and reduced levels of Mitsuokella and Eubacterium ruminantium. Dietary interactions, e.g., mango, lettuce, and carrot, influenced specific taxa (p<0.05).

**Conclusion:**

This study pioneers gut microbiota characterization in AD and MCI in the Dominican Republic, identifying microbial alterations in cognitive impairment and highlighting regional dietary and ethnic factors. Longitudinal and multi-omics studies are warranted to clarify causality and therapeutic potential.

## RESUMEN

La enfermedad de Alzheimer (EA) y el deterioro cognitivo leve (DCL) son importantes contribuyentes de demencia, con creciente prevalencia en Latinoamérica. La evidencia sugiere influencia de alteraciones microbianas intestinales en la neurodegeneración, con limitados datos en hispanohablantes.

**Objetivo:**

Caracterizar la microbiota intestinal en individuos con EA, DCL y controles en República Dominicana, explorando asociaciones clínicas, demográficas y dietéticas.

**Métodos:**

Estudio prospectivo-traslacional con 88 participantes de ≥60 años. Se realizaron evaluaciones clínicas, cognitivas y funcionales. Las muestras fecales se analizaron mediante secuenciación 16S rRNA. Se evaluaron diversidad alfa y beta, taxonomía y abundancia diferencial mediante QIIME2 y R. Las influencias dietéticas se analizaron con PERMANOVA.

**Resultados:**

No se observaron diferencias significativas en diversidad alfa (índice Shannon 4–5, índice Simpson 0.94–0.99, p>0.05) ni beta (p>0.05) entre grupos. Firmicutes (51.9%) y Bacteroidota (34.1%) fueron predominantes. DCL y EA mostraron mayor abundancia de Desulfobacterota (0.54% y 0.61% vs. 0.34% en controles; p < 0.05). La relación Firmicutes/Bacteroidota fue menor en hombres con DCL (1.09) comparada con controles y EA (1.70). DCL y EA se asociaron con niveles elevados de Bilophila, Odoribacter y Parabacteroides (p<0.05), y reducidos de Mitsuokella y Eubacterium ruminantium. Fueron identificadas influencias de interacciones dietéticas (mango, lechuga y zanahoria) en taxones específicos (p<0.05).

**Conclusión:**

Este estudio caracteriza por primera vez la microbiota intestinal en EA y DCL en República Dominicana, identificando alteraciones asociadas con neurodegeneración y la influencia de factores regionales. Se requieren estudios longitudinales y multi-ómicos para esclarecer causalidad y potencial terapéutico.

## INTRODUCTION

Alzheimer's disease (AD) is one of the leading causes of dementia worldwide, currently affecting more than 55 million people, with projections indicating a near doubling by 2050, driven by population aging^1^. This neurodegenerative condition is characterized by the accumulation of β-amyloid plaques, the formation of neurofibrillary tangles of the Tau protein, and progressive neuroinflammation, processes that lead to cognitive decline and functional disability^2,3^. Mild cognitive impairment (MCI), considered an intermediate stage between normal aging and dementia, has gained relevance due to its potential progression to AD^4^. However, the underlying pathogenic mechanisms of AD remain incompletely defined^5^.

In recent years, the gut microbiota has emerged as a key modulator of the host's immunological, metabolic, and neurological functions^6^. Established data suggest that alterations in the intestinal microbial composition could contribute to age-related cognitive decline and the progression of neurodegenerative diseases such as AD^7^. Furthermore, a connection has been established through the gut-brain axis, whereby intestinal dysbiosis can induce neuroinflammation and alter brain function^8^. Quantitative and qualitative alterations in the gut microbiota in patients with AD have been reported, highlighting increased abundance of specific taxa (e.g., Bacteroides and Verrucomicrobia)^9^ and decreased abundance of Firmicutes and Proteobacteria^10,11^. These findings suggest that intestinal dysbiosis may play a significant role in the pathophysiology of these conditions; however, inconsistent results persist across studies^12^.

Additionally, it has been documented that factors such as sex, diet, geographic environment, and, particularly, ethnicity influence the composition of the gut microbiota^13-15^. Recent studies have shown significant ethnic group variations in different clinical conditions, such as autism, cancer, irritable bowel syndrome, and obesity^16-20^. These differences could have important implications for diagnosis and personalized treatment, especially in neurodegenerative diseases. Studies on this topic have been conducted in high-income countries (the United States, Japan, China, Korea, and some European countries). An advanced search in the United States National Library of Medicine (PubMed), Scopus, Scientific Electronic Library Online (SciELO), and Google Scholar, using Medical Subject Headings (MeSH) terms, keywords, and geographic filters, revealed the absence of research focused on the gut microbiota and its relationship with MCI or AD in Latin America. This lack of representation limits knowledge of the specific characteristics of these populations, in which dietary, environmental, and genetic factors may substantially influence microbial composition^21^. Moreover, the high prevalence of metabolic disorders and distinctive dietary patterns in Latin America could shape microbiota profiles relevant to neurodegeneration pathways^22-24^.

The Dominican Republic, as a middle-income country with a diverse and understudied population, represents a strategic opportunity to explore these interactions. The present study aims to characterize the gut microbiota in participants with MCI, AD, and healthy controls using high-resolution sequencing techniques to assess its composition and diversity. This research seeks to provide local evidence on the mechanisms involved in cognitive decline and to evaluate the potential of the microbiota as a biomarker and therapeutic target in neurodegenerative diseases.

## METHODS

A prospective translational study was conducted to characterize the gut microbiota in participants with MCI, AD, and healthy controls. The study protocol was approved by the Bioethics Committees of the Faculty of Health Sciences of the Pontificia Universidad Católica Madre y Maestra (PUCMM) (COBEFACS-ext-004-2-2021-2022) and by the National Bioethics Council in Health (CONABIOS) of the Ministry of Public Health of the Dominican Republic. Informed consent was explained to participants, including the benefits and risks of participating in the study, in accordance with the Helsinki Declaration. All participants signed a consent form, and for patients with advanced dementia, the consent was signed by a designated family member or legal guardian.

### Sample size and recruitment

A non-probabilistic sample of 90 participants was planned by expert consensus; two were excluded due to sample contamination. A total of 88 stool samples were analyzed. Recruitment was conducted between January and December 2023 at the Geriatrics clinic of Clínica Corominas, a university-affiliated hospital of PUCMM. Patients and their relatives attending the outpatient geriatric clinic were informed by the treating geriatrician about the study objectives and procedures. Individuals expressing interest were referred to the principal investigator, who provided a detailed explanation of the study protocol, provided the consent form, and addressed any questions. A separate visit was scheduled for clinical assessment and formal consent signing by the participant and, when applicable, a family member or legal representative.

### Clinical and functional assessments

Sociodemographic data (age, sex, educational level), medical history (hypertension, diabetes, dyslipidemia, ischemic heart disease, hysterectomy), and habits (tobacco, alcohol, coffee, marijuana) were collected, along with medication use. Cognitive assessment was performed with the Montreal Cognitive Assessment (MoCA), using the basic version in participants with ≤12 years of education^25^ and the standard version in the rest^26^. Neuropsychological assessments used validated measures for Spanish-speaking individuals from the Taub Institute at Columbia University^27^. Functionality was assessed using the Lawton and Brody Instrumental Activities of Daily Living Scale^28^. The diagnosis of MCI was based on Petersen's criteria^29^ and that of dementia on the National Institute on Aging — Alzheimer's Association (NIA-AA) criteria^30^. Severity was determined using the Clinical Dementia Rating (CDR) scale^31^. The Geriatric Depression Scale (GDS) was also applied to rule out depression^32^. Participants were classified according to cognitive status: controls had a MoCA≥25 and CDR=0, participants with MCI had a MoCA 14–24 and CDR=0.5, and participants with AD had a MoCA<14 and CDR=1–2. Participants underwent brain MRI and laboratory analysis (complete blood count, lipid profile, vitamin B12, folic acid, TSH, and free T4). Physical activity level, type, frequency, and minutes per week; food type and frequency; and medical physical examination, including blood pressure measurement, body mass index (BMI), abdominal circumference in cm, and complete neurological examination, were performed. The data collection form included a specific section for recording habitual medication use. In addition, a structured food frequency questionnaire was designed that included the main food groups (dairy products, beverages, fish and seafood, meats, processed meats, eggs, cereals, legumes, bread, tubers, vegetables, fruits, fats, nuts, and sugars). Consumption frequency was classified using a four-category ordinal scale: 0=never or occasional, 1=weekly consumption, 2=biweekly consumption, and 3=monthly consumption. Participants were included or excluded according to the following criteria:

### Healthy controls

#### Inclusion criteria

- No history of neurological or psychiatric disease;
- absence of cognitive impairment on detailed neuropsychological evaluation;
- MoCA score ≥25;
- CDR score of 0;
- age ≥60 years;
- oral feeding without the use of feeding tubes or texture-modified diets;
- provision of written informed consent.

#### Exclusion criteria

- Diagnosis of mild cognitive impairment or dementia;
- chronic or prolonged use of antibiotics or laxatives;
- history of gastrointestinal disorders affecting gut microbiota, including irritable bowel syndrome, functional dyspepsia, centrally mediated abdominal pain syndrome, or inflammatory bowel disease;
- immunological diseases or active cancer;
- current treatment with chemotherapy or radiotherapy;
- use of special diets; and
- history of cerebrovascular accident, alcoholism, psychiatric disorders, or gastrointestinal surgery.

### Mild cognitive impairment

#### Inclusion criteria

- Diagnosis of mild cognitive impairment according to Petersen's criteria;
- CDR score of 0.5;
- MoCA score between 14 and 24;
- preserved basic activities of daily living;
- age ≥60 years;
- oral feeding without the use of feeding tubes or texture-modified diets; and
- provision of written informed consent.

#### Exclusion criteria

- Diagnosis of dementia according to NIA-AA criteria;
- chronic or prolonged use of antibiotics or laxatives;
- history of irritable bowel syndrome, functional dyspepsia, centrally mediated abdominal pain syndrome, or inflammatory bowel disease;
- immunological diseases or active cancer;
- current treatment with chemotherapy or radiotherapy;
- use of special diets; and
- history of cerebrovascular accident, alcoholism, psychiatric disorders, or gastrointestinal surgery.

### Alzheimer's disease

#### Inclusion criteria

- Diagnosis of sporadic or familial Alzheimer's disease according to NIA-AA criteria;
- CDR score between 1 and 2, indicating mild to moderate dementia;
- MoCA score <14;
- Barthel Index ≥90;
- age ≥60 years;
- oral feeding without the use of feeding tubes or texture-modified diets; and
- provision of written informed consent by the participant and/or a responsible family member.

#### Exclusion criteria

- Chronic or prolonged use of antibiotics or laxatives;
- history of irritable bowel syndrome, functional dyspepsia, centrally mediated abdominal pain syndrome or inflammatory bowel disease;
- immunological diseases or active cancer;
- current treatment with chemotherapy or radiotherapy;
- use of special diets; and
- history of cerebrovascular accident, alcoholism, psychiatric disorders, or gastrointestinal surgery.

### Sample collection and microbiome analysis

Stool samples were collected by each participant using the DNA/RNA Shield™ tube (Zymo Research, USA) and processed at Darwin Bioprospecting Excellence, SL (Valencia, Spain), in accordance with International Human Microbiome Standards (IHMS). DNA extraction was performed with the Mag-Bind^®^ Universal Pathogen 96 Kit (Omega Bio-Tek, USA), and quantification was performed with Qubit dsDNA High Sensitivity (Invitrogen, USA). Library preparation followed the standard Illumina protocol, and sequencing was performed on the Illumina HiSeq platform (2x300 bp), as described by Satari et al.^33^

### Bioinformatic and statistical analysis

Sequences were processed using Quantitative Insights Into Microbial Ecology Version 2 (QIIME2)^34^. Quality assessment was conducted with Divisive Amplicon Denoising Algorithm 2 (DADA2), and amplicon sequence variants (ASVs) were taxonomically assigned using classify-Sklearn in conjunction with the SILVA v138 database^35^. For microbial ecology and statistical analysis, the following packages were employed: R Phyloseq^36^ and Vegan^37^. Data normalization was performed using the total sum normalization (TSS) method, and visualizations were created with KronaTools^38^. Differential abundance analysis using normalized counts was carried out with LEfSe^39^ and the microbiomeMaker package^40^, utilizing the parameters: kw_cutoff=0.05, lda_cutoff=2, norm="rarefy", and transform="identity." More than 130 thousand reads per sample were confirmed, with an average of 220,198±2,693 reads. Finally, MaAsLin2^41^, which incorporates false discovery rate (FDR) correction using the Benjamini-Hochberg procedure, was used to identify differences between taxa. The following parameters were applied: min_abundance=0, min_prevalence=0.1, normalization="TSS", transform="LOG", analysis_method="LM", correction="BH", and standardize=FALSE.

## RESULTS

### Demographic and clinical characteristics

The characteristics of participants with MCI, AD, and controls are presented in Table 1. No statistically significant differences were found between the three groups regarding gender (p=0.489), hypertension (p=0.366), diabetes mellitus (p=0.162), active smoking (p=0.413), smoking history (p=0.652), and BMI (p=0.238). Participants with MCI and AD were older than the controls, with a significant difference (p=0.018). Current alcohol use and previous alcohol consumption were more frequent in the control group (p=0.003 and p=0.005, respectively). Participants with MCI and AD were more sedentary than controls (p=0.012).

**Table 1:** Demographic characteristics of the study participants.

Variable (%)	Total (n=83)	Control (n=26)	MCI (n=32)	Dementia (n=25)	p-value
Female gender	70.5	22.7	28.4	22.7	0.489
Mean age (SD)	73.35 (7.33)	69.23 (4.83)	76.16 (6.56)	73.93 (8.41)	0.018
Hypertension	71.6	18.2	28.4	25.0	0.366
Diabetes Mellitus	20.5	4.5	11.4	4.5	0.162
Current smokers	1.1	0	1.1	0	0.413
Ex-smokers	12.5	2.3	5.7	4.5	0.652
Current alcohol consumption	27.3	14.8	9.1	3.4	0.003
Past alcohol consumption	11.4	8.0	0	3.4	0.005
Sedentarism	65.9	12.5	27.3	26.1	0.012
Light activity	28.4	11.4	9.1	8.0	
Moderate or strenuous activity	5.7	5.7	0	0	

### Alpha and beta diversity of the gut microbiome

**Figure 1:**
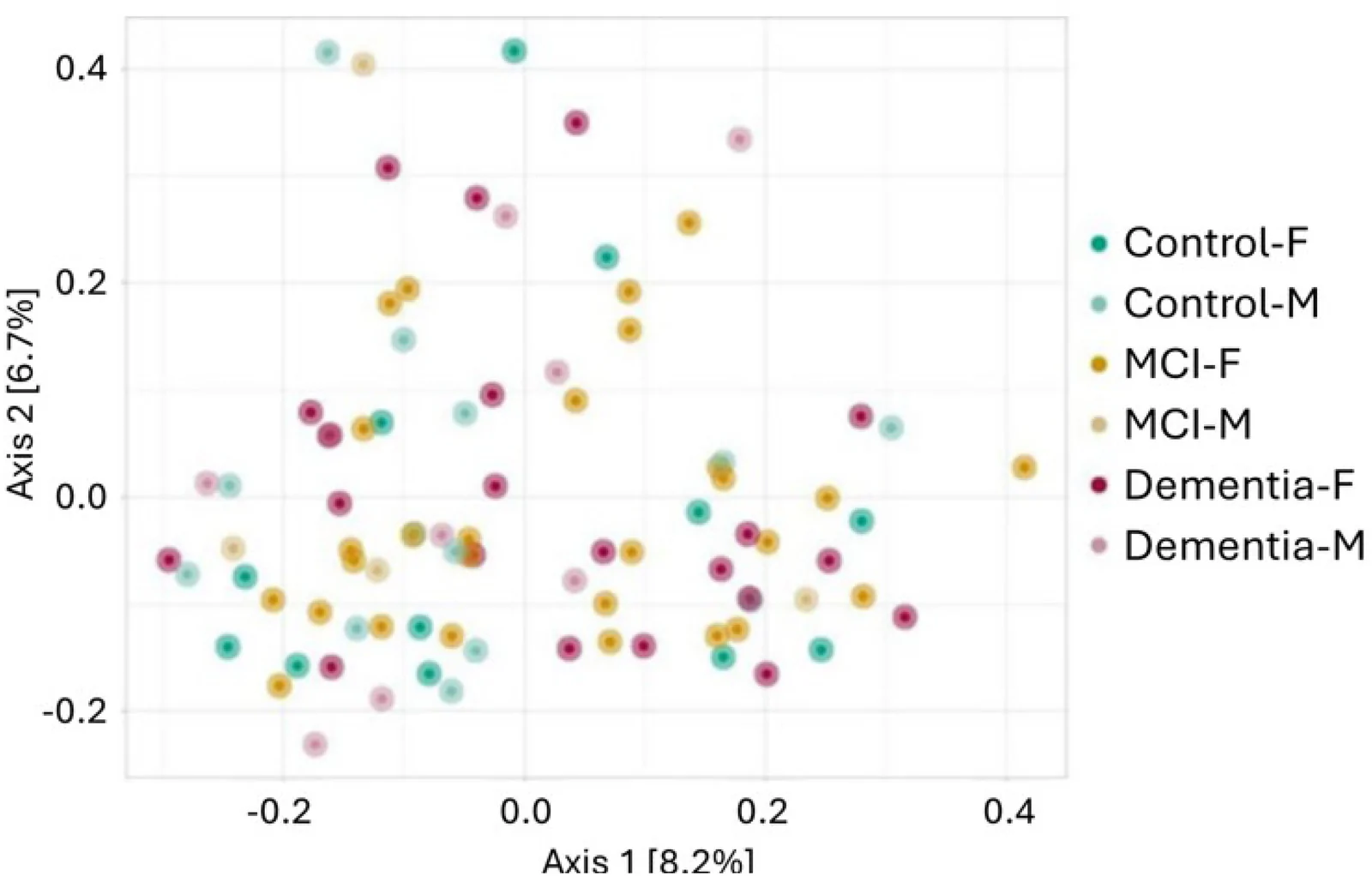
β Diversity at the level of amplicon sequence variants between different patient groups.

Abbreviations: MCI, mild cognitive impairment; F, female; M, male.

Alpha diversity (α-diversity) was assessed by the number of amplicon sequence variants (ASVs), Shannon indices, and Simpson indices. No significant differences were observed between groups or by sex. Species richness ranged from 300 to 750 ASVs, with Shannon values between 4 and 5 and Simpson values between 0.94 and 0.99. Beta diversity (β-diversity), analyzed by Principal Coordinates Analysis (PCoA) of Bray-Curtis dissimilarity matrices, showed no evident clustering by study group or sex (Figure 1). PERMANOVA analysis confirmed the absence of significant differences (p>0.05). Clinical variables such as age, BMI, hypertension, and diabetes also did not influence microbial composition. However, the interaction between patient type and consumption of certain foods (lettuce, banana, grape, and olive oil) was significant (p<0.05). Likewise, the interaction of consumption of longaniza (Dominican sausage), lettuce, carrot, and mango varied according to the degree of cognitive impairment.

### Alterations in intestinal microbial composition

**Figure 2:**
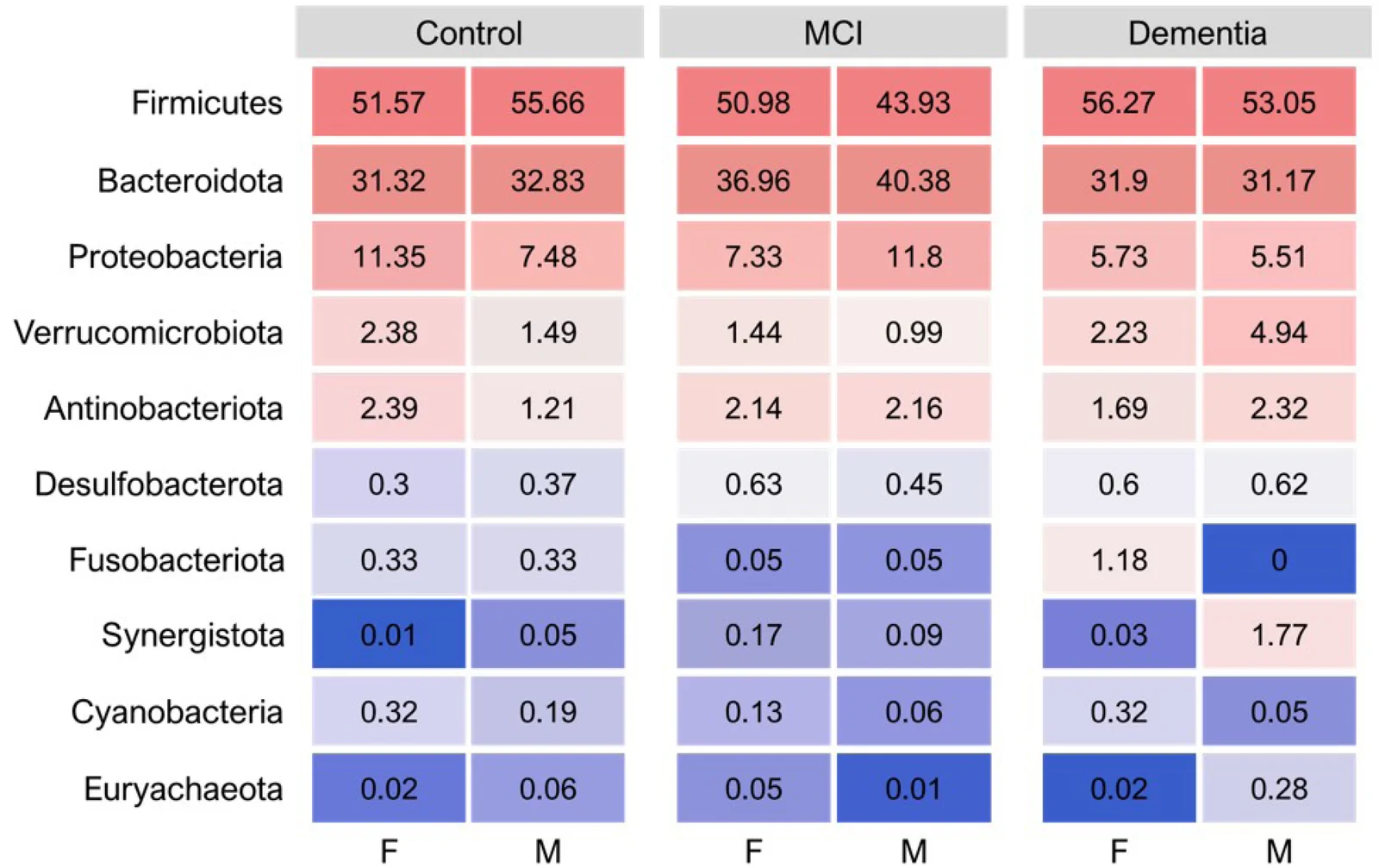
Heat map illustrating the average relative abundance (%) at the phylum level, grouped by patient type and sex.

Abbreviations: MCI, mild cognitive impairment; F, female; M, male.

**Figure 3:**
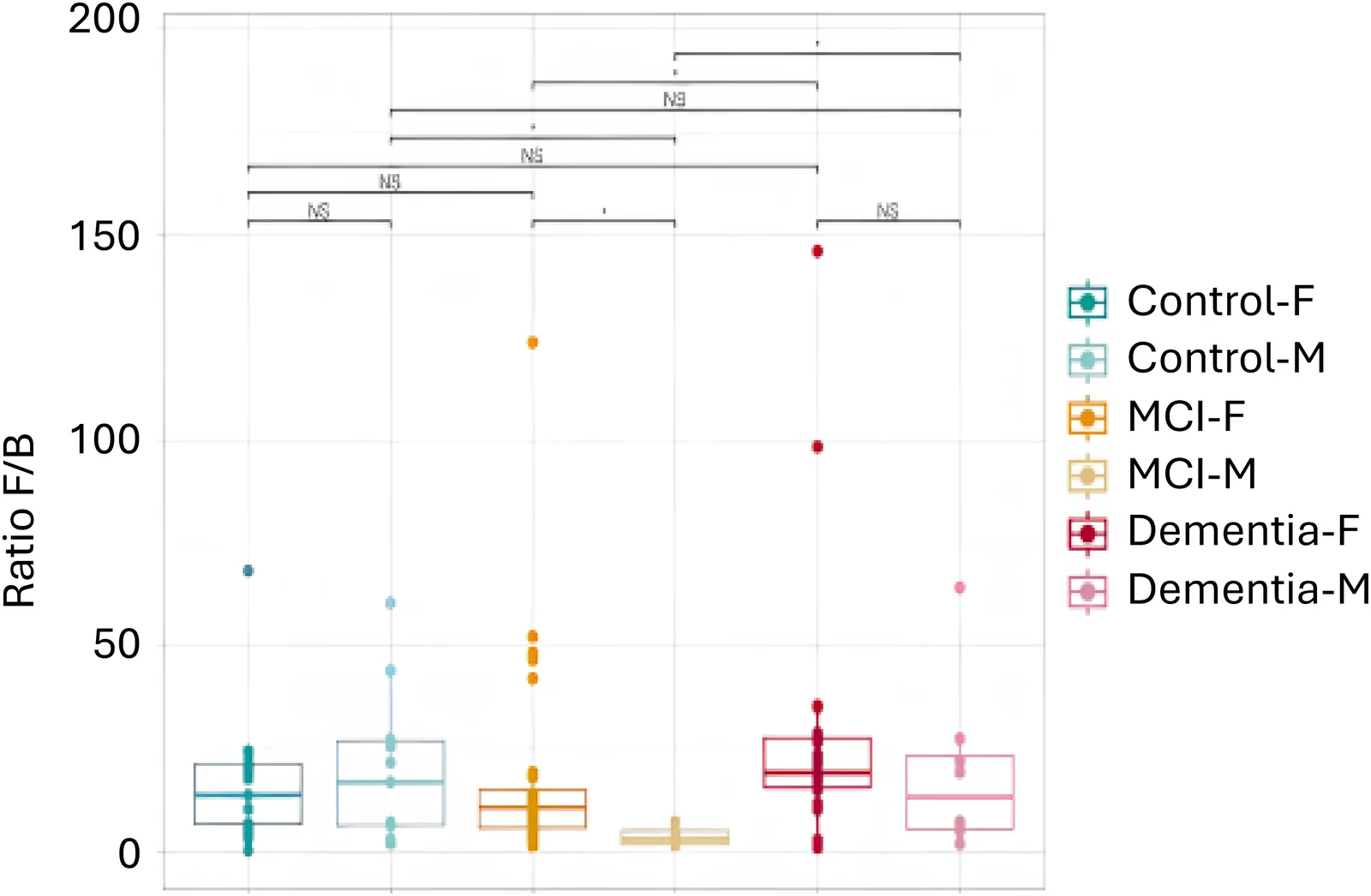
Firmicutes/Bacteroidota ratio values grouped by group and sex.

Abbreviations: MCI, mild cognitive impairment; F, female; M, male.

Taxonomic analysis revealed that the microbiome was dominated by Firmicutes (51.9%) and Bacteroidota (34.1%), followed by Proteobacteria (8.2%), Verrucomicrobiota (2.3%), Actinobacteriota (2.0%), and Desulfobacterota (0.5%) (Figure 2). Notably, patients with MCI and AD showed a higher abundance of Desulfobacterota (0.54 and 0.61%, respectively) compared to controls (0.34%) (p<0.05). Additionally, a higher proportion of Firmicutes was found in AD (54.7%) and Bacteroidota in MCI (38.7%). The Firmicutes/Bacteroidota (F/B) ratio was lower in men with MCI (1.09) compared to controls and AD patients (both 1.70). In women, the F/B ratio was lower in MCI (1.38) compared to AD (1.76), with a trend toward higher values in controls (1.65) (Figure 3).

**Figure 4:**
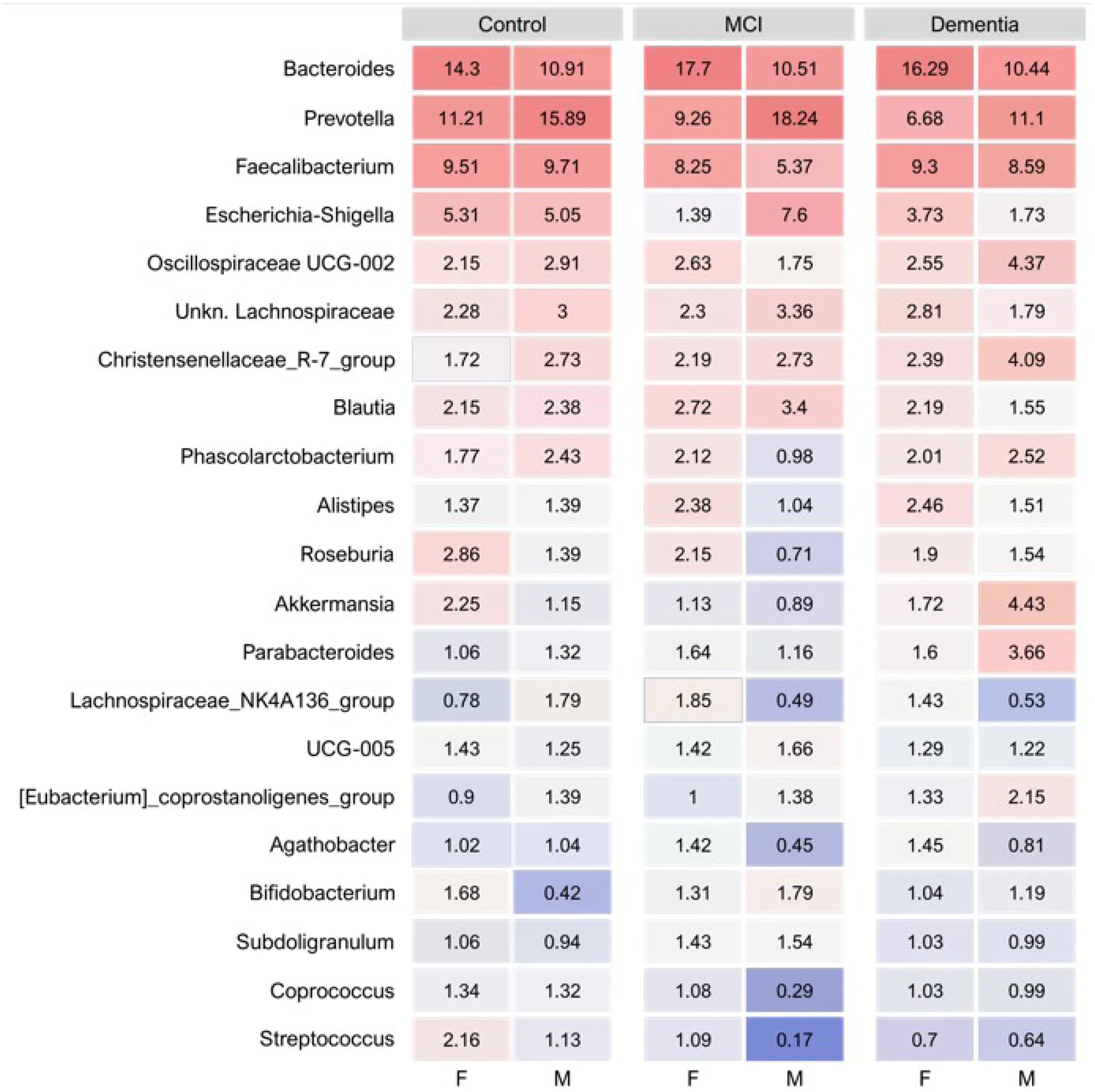
Taxonomic distribution (in average relative abundance) at the level of gender, by patient and sex.

Abbreviations: MCI, mild cognitive impairment; F, female; M, male.

At the genus level, the predominant taxa were Bacteroides (13.4%), Prevotella (12.1%), Faecalibacterium (8.5%), and Escherichia-Shigella (4.1%), which, together with 17 other genera, accounted for 66.8% of the total (Figure 4). Specific differences were observed, including a reduction in Mitsuokella in MCI compared to controls. Butyrate producers (Butyricimonas, Hungatella, [Eubacterium] hallii) were more abundant in MCI. [Eubacterium] ruminantium was less frequent in MCI. Bilophila increased in MCI and AD. In AD, increases in Odoribacter, Oscillibacter, Coprobacter, and Parabacteroides were observed.

### Influence of diet on the microbiota

Interaction analysis by PERMANOVA showed that consumption of longaniza, lettuce, carrot, and mango influenced the groups differently. Among consumers of mango, lettuce, and carrot, controls had higher abundances of Mitsuokella and [Eubacterium] ruminantium. In contrast, patients with MCI or AD showed increases in Parabacteroides, Butyricimonas, Bilophila, and Odoribacter.

## DISCUSSION

This study constitutes the first characterization of the gut microbiota in AD and MCI in the Dominican Republic and, in general, one of the first regional approaches in Latin America. Given the growing burden of dementia in the region, driven by population aging, and its implications for health systems, having local data is especially relevant^42,43^. In our samples, no significant differences in α-diversity or β-diversity were identified between controls, MCI, and AD. This finding is consistent with previous mixed results: while some studies have reported global diversity changes in AD, others have found no global differences, and instead have reported specific alterations by taxa^33,34,39,44^. In our cohort, the most consistent signals emerged precisely at that level: shifts in specific phyla and genera.

At the phylum level, we observed higher Desulfobacterota in MCI and AD compared to controls, which is biologically plausible given its involvement in pro-inflammatory sulfate-reduction pathways and its association with amyloidogenic and microglial processes in experimental models^45^. The Firmicutes/Bacteroidota (F/B) ratio showed a reduced pattern in MCI (especially in males) compared with controls and AD; this pattern, although not uniform in the literature, partially aligns with studies reporting lower Firmicutes and higher Bacteroidetes in MCI/AD^34,39^. At the genus level, we detected lower levels of Mitsuokella and [Eubacterium] ruminantium in MCI compared to controls; these genera have been linked to neurotransmitter pathways and intestinal barrier homeostasis, so their decreases could be related to neurocognitive dysfunctions^46^. Notably, butyrate producers (Butyricimonas, Hungatella, [Eubacterium] hallii) were more abundant in MCI. However, some studies have reported reductions in butyrate in cognitive impairment (e.g., Butyricimonas in MCI)^47^. Our finding could reflect an early compensatory response aimed at maintaining intestinal barrier integrity and modulating neuroinflammation^46,48^. Likewise, linked to inflammation, Bilophila increased in MCI and AD; and in AD, increases in Odoribacter, Oscillibacter, Coprobacter, and Parabacteroides stood out, in line with pro-inflammatory profiles described in other series^34,39,49^.

Taken together, these results support the gut-brain axis hypothesis, whereby intestinal microbial alterations can modulate neuroinflammation and the cognitive phenotype through metabolites (e.g., SCFA, bile acids), immune pathways, and neural mechanisms (vagus, ENS)^44,48,50^ — in particular, SCFAs, such as butyrate, exert epigenetic, intestinal barrier-reinforcing, and anti-inflammatory effects with potential neuroprotective effects^46,48^. Data from clinical cohorts link specific taxa with neurodegeneration and Aβ biomarkers^49,51^, while in animal models dysbiosis has been observed to precede amyloidosis and microglial activation^45^. This is complemented by emerging evidence on microbial modulation interventions, from diet to fecal transplantation, with favorable signals in neuroinflammation and cognitive performance in early stages^52,53^.

A crucial dimension for interpreting global heterogeneity is the ethnic-dietary and environmental context. Studies in the US, Europe, and Asia show that diet, genetics, and environment condition the microbial profile and could explain discrepancies between cohorts^52^. For example, in the Japanese population, higher Bacteroides in dementia and correlations with neurodegeneration markers have been reported^51^, while in Shanghai, increases in Erysipelotrichales and Saccharimonadales associated with APOE ε4 and clinical severity have been described^33^. In Korea, specific differences by race/ethnicity have been documented even in MCI^54^. Our findings, obtained in a Caribbean cohort with local dietary patterns, precisely provide that regional nuance lacking in the literature.

### Strengths and limitations

This study has several strengths and limitations that are essential to consider when interpreting the findings. The strengths include the comprehensive characterization of the gut microbiota in individuals with AD and MCI in the Dominican Republic, an understudied region in this context. Furthermore, the study employed high-resolution sequencing and a standardized bioinformatic pipeline, thereby ensuring high data quality and reliability. Additionally, the inclusion of dietary variables enabled detection of interactions between patient type and diet, providing valuable insights into the relationship between the gut microbiota and cognitive decline. However, the study also has several limitations. Notably, the absence of AT(N) biomarker-based AD phenotyping may limit the understanding of the relationship between the gut microbiota and AD pathology. Another limitation was the lack of APOE genotype data, a significant risk factor for AD that may influence the gut microbiota. Furthermore, the sample size may be limited, potentially affecting the detection of subtle effects or the ability to conduct additional stratified analyses. Moreover, within the control group, four participants were spouses living in the same household. We acknowledge that cohabitation may partially influence gut microbiota composition due to shared environmental factors. However, we consider that the inclusion of these participants is unlikely to have compromised the overall validity of the findings, given their small proportion relative to the total sample and the strict application of exclusion criteria, particularly those related to gastrointestinal disease, antibiotic use, and special diets.

### Implications and future directions

The findings support the involvement of the gut microbiota in MCI and AD, suggesting specific microbial signals emerge in prodromal stages. Future studies should prioritize longitudinal, multi-omics approaches to clarify causal directionality and assess the biomarker value of the microbiota. Including biological AT(N) phenotyping and APOE genotype data will provide a more comprehensive understanding of the relationship between the gut microbiota and AD pathology. Standardizing dietary patterns will help reduce variability, while exploring microbial modulation strategies may offer new avenues for slowing cognitive decline. Considering the ethnic and cultural context will be crucial for clinical translation in Latin America.

In conclusion, this study provides the first comprehensive characterization of the gut microbiota in individuals with MCI and AD in the Dominican Republic, and one of the first such analyses in the Caribbean and Latin America. The findings highlight the involvement of gut microbial alterations in the pathophysiology of cognitive decline and underscore the influence of regional, dietary, and ethnic factors on the microbiome. The gut microbiota holds promise as a key player in the prevention and management of neurodegenerative diseases; therefore, prioritizing research in this area is crucial to advancing our understanding of causal relationships, evaluating the biomarker potential of the microbiota, and exploring targeted interventions to prevent and manage Alzheimer's disease spectrum in Latin America.

## Data Availability

The datasets have been deposited in the NCBI Sequence Read Archive (SRA) under BioProject ID: PRJNA1392028.
